# The Foreign Journals: Pathology, Practical Medicine, and Therapeutics

**Published:** 1840-10

**Authors:** 


### PATHOLOGY, PRACTICAL MEDICINE, AND THERAPEUTICS.

### *On Cramp of the Tongue.* By Dr. Hoffman, of Suhl.

A person suffering from chronic gout came to me in great distress for my immediate assistance, saying that since the previous day he had had four attacks of such severe cramp of the tongue, that it had been completely rolled inwards towards the frenum. The frenum itself also was extremely painful. During the attack which lasted from ten to fifteen minutes, he was unable to speak, and could only utter hollow expressions of pain; he felt also considerable constriction of the chest. As he was a full-blooded man I ordered him to be largely bled, to abstain rigidly, to take nitre with ipecacuan and aconite, and to use as a gargle an emulsion with laudanum. He had only one slight attack after the use of these measures.

*Medicinische Zeitung. June* 3, 1840.

---

### *On the Pain of the Back in Intermittent Fever.* By Dr. Grossheim.

This paper contains the results of observations made in fifty cases of intermittent fever, for the purpose of testing the opinions of Dr. Kremer (noticed in our Fifteenth Number, Vol. VIII., p. 67), who had stated that a painful sensation is a constant and pathognomic sign of intermittent fever, on pressure over the first dorsal vertebra and the adjacent parts of the spine, and that there is a constant relation between the severity of this symptom and of the general disease. The following are the results which Dr. Grossheim has obtained, and which seem to confirm Dr. Kremer's statements, although they had been contradicted, as we formerly stated, by the experience of other observers: 1. Pain on pressure on some part of the spinal column is a constant symptom of intermittent fever, except in those cases in which the ligaments of the vertebræ have become by age or disease so rigid that they will not yield to pressure. 2. There is no definite locality to this pain; it may be situated in any part of the column, but is most frequent in the middle of the dorsal portion, especially in quotidian intermittents. 3. The extent of the pain also varies considerably; one or two vertebræ only may be tender; and the pain rarely occupies the space of more than five or six; it may also be situated at distant parts, with intervals in which none is excited by pressure. 4. The intensity of the pain is equally variable. Sometimes it was so severe that the slightest touch of one of the spinous processes produced severe suffering; but sometimes violent pressure was required to detect it. Among those vertebræ that excited pain when pressed, the middle one was commonly the most sensitive; and in those above and below it, the tenderness gradually decreased. 5. The pain was more severe during the paroxysms than in the intermissions. When the severity of the fever diminished or the tendency to its returns grew less, the severity and the extent of the pain in the back also decreased; but the complete removal of the fever was not always accompanied by the entire loss of the pain, which often continued in a modified degree after the fever had ceased to return, and remained the longer the more severe it had previously been. 6. Complications of the intermittent fever did not appear to have any influence on the pain; it continued when the character of the fever was altered either for the better or for the worse, and it returned in cases of relapse.

From observing the constant existence of this symptom, the author was induced to try what would be the effect of remedies that would tend to correct the local excitement that seemed to exist. He relates very briefly five cases, in which eight or ten leeches were applied over the spine in the situation where pressure gave the most pain. In four of these no other remedy was required; the pain ceased in a few days, and there was no recurrence of the febrile paroxysm.

*Medicinische Zeitung. June* 3, 1840.

*On the Presence of Iodine in Cod-liver Oil.* By L. GMELIN.

[THE source of the therapeutic utility of this very nauseous medicine is in a great measure demonstrated by the following observations of Professor Gmelin.]

I had announced, says Professor Gmelin, that it was impossible to detect iodine in two kinds of cod-liver oil, of which one had a clear and the other a brown colour; and I had left it undecided whether the iodine found by other chemists had proceeded from the iodate of soda employed in their experiments, or whether certain samples of the oil did really contain iodine. The following researches will show that the genuine oil does clearly contain iodine, and that the examples of it which I first examined were spurious:

By treating sixty grammes of pure cod-liver oil, from Bergen in Norway, by Hausmann's method, I obtained by solution in alcohol a saline mass, which acted in the following manner: Its aqueous solution, mixed with starch and diluted sulphuric acid, gave a violet colour, which immediately disappeared on adding oil of vitriol, and was exchanged for a yellow hue. The same solution gave with starch and chlorhydric acid a violet colour, which soon disappeared on the addition of chlorate of potash. The experiment was repeated with 750 grammes of the same oil. The results were the same; only that in consequence of the greater quantity, the colouring of the starch was much more intense, and was not so promptly destroyed by the oil of vitriol or the chlorate of potash. Carburet of sulphur agitated with the aqueous solution of the saline mass with the addition of diluted chlorhydric or sulphuric acid was coloured violet; and when a portion of the same saline mass was thrown into a mixture of peroxyde of manganese and moderately diluted sulphuric acid, which had been previously heated in a glass tube, the violet vapours of iodine were immediately seen rising and staining starch-paper blue.

These experiments leave no doubt of the presence of iodine in this cod-liver oil. On the authority of M. Tiedemann, a merchant at Bremen who has an intimate knowledge of the article, Prof. Gmelin says that there are in commerce four kinds of genuine cod-liver oil. The oil is melted by exposing the livers to the sun in casks which are placed upright, and divided into three parts by moveable boards placed one above the other. The clearest oil, and that which is most fit for medicinal purposes is that which floats to the top; the next layers are coarser, and the lowest are quite brown. The refuse of the casks forms a deep-coloured and thick oil, which is used in the manufacture of leather.

[In the Dublin Journal for July last, there is a valuable paper by Mr. Donovan, pointing out the great desideratum of rendering this oil palatable. He says this is effected by using a temperature in its expression not exceeding 192°.]

*Bulletin de Thérapeutique. Mai,* 1840.

---

*The Rest-harrow (Ononis Spinosa, or O. Arvensis,) a Remedy for Rheumatism.* By Dr. ASCHERSON, of Berlin.

OF this, as of many other remedies for chronic rheumatism, little more can be said than that a respectable author adds his testimony to the correctness of popular belief. Dr. Ascherson first heard of the virtues of rest-harrow (a common weed in this country as well as in Germany) from a washerwoman, and being convinced that it cured her, he tried it in several other cases and found it surprisingly beneficial. It was not invariably successful, but it never did harm, and cured many cases that had long resisted other means. The form of administration is a concentrated decoction of the fresh herb with its roots, or of the roots and stems dried; and a quart of the decoction must be taken daily. Its immediate effect is powerfully diuretic.

*Casper's Wochenschrift. June* 6, 1840.

*On the Production of Chronic Ventricular Hydrocephalus, in consequence of the pressure exercised by tuberculous masses of the Cerebellum on the Straight Sinus.* By M. F. BARRIER.

[THE object of this paper is sufficiently evident from its title; two cases are carefully narrated which at least prove coincidence of the two anatomical states referred to, while analogy favours the notion that the obstruction to the return of the blood through the straight sinus may have been the cause of the local dropsy. These cases the author himself candidly admits may be of very rare occurrence; nevertheless, as we agree with him in regarding the point as one of no mean interest, we extract some of his general remarks respecting it.]

The anatomical conditions capable of explaining the production of this form of hydrocephalus are: 1, the presence of the tuberculous mass in the *middle lobe* of the cerebellum; 2, its forming a considerable projection on its superior surface, so as to push the tentorium cerebelli upwards and exercise pressure on the straight sinus. Other conditions conducing to the same result might also be encountered; thus inflammation might lead to adhesion of the tumour with the tentorium, and gradually spreading to the interior of the sinus, determine the formation of a coagulum which in its turn would oppose the circulation of the blood returning through the venæ Galeni from the ventricles: these veins might also undergo pressure on their egress from the fissure of Bichat. It is evident that obliteration of these vessels, as well as of the straight sinus, can only produce one species of hydrocephalus, that of the ventricles, as the veins springing from the walls of these cavities form a system apart.

There may, it is true, be found in authors, numerous cases of tuberculous growth in the cerebellum unattended with ventricular dropsy; but it will be perceived in all those related with detail, that the tumour was seated either in the lateral lobes or at the inferior surface of the cerebellum, or that, if developed at the upper surface, it did not protrude upwards sufficiently to exercise pressure on the straight sinus. On the other hand, chronic non-congenital hydrocephalus is frequently seen without coincident tumour in the cerebellum.

*Gazette Médicale de Paris.* No. 17. *Avril,* 1840.

---

*Coincident Occurrence of Variola, Modified Variola, and Varicella in the same Individual.* By Dr. BRUNZLOW, of Brandenburg.

ONE of the author's family, a child a year and a half old with a very irritable skin, who had been vaccinated when a few months old with lymph taken directly from the arm of a healthy child, and who had had five regular vesicles on the two arms, was affected during its infancy with various exanthemata. It had scarcely recovered from an attack of urticaria when the diseases mentioned above began to appear, though the child had not been in contact with any one affected by them. The smallpox eruption occurred in a measure regularly; it commenced on the face and finished on the extremities, so that within a few days the whole body was covered with an innumerable quantity of pustules.

When the eruption was completed, and when, according to the general rule, no new pocks ought to have broken out, there appeared both on the face and on the trunk and extremities, and between the vesicles that were already formed and were passing through their regular course, a number of others far smaller, paler, and possessing less activity, which both in their form and in their course were very different from those that first appeared, and which from all their characters could be regarded only as varicella.

The vesicles first mentioned went on in their development, increased in size, became filled with a serous fluid, presented depressions at their centres, and became surrounded with a red areola; but the author observed that after the fourth day of their eruption, the majority of them did not go on to suppuration. Their redness diminished, the lymph which they contained dried up, and after a few days they were covered with crusts. They presented evident examples of modified smallpox, the varioloid disease.

Several of the vesicles of the first eruption, however, did not dry up, but went on further in their development. They filled with pus, their areolæ reddened and were vividly inflamed, and after a few days they acquired the form of genuine and perfect smallpox. Their number, however, was not considerable; there were few on the face but they were more numerous on the abdomen and on the inner side of the forearm and thigh.

The fever accompanying the eruption, which did not diminish on the drying of the varioloid eruption, increased with the progressive suppuration of these last pustules, and became rather severe.

The varicella, while the variolous eruption was suppurating and the varioloid was drying, had not yet finished coming out; and new vesicles of it kept constantly appearing among those of the other diseases, and after lasting a few days and filling with a watery lymph, dried and became covered with a fine light scurf.

At last when the variolous eruption had completed its stage of suppuration and passed into that of drying, and when the desquamation of the varioloid was finished, the fever diminished, no more vesicles of varicella appeared, the patient became more lively, and regained his appetite, and soon after was completely restored to health. The diseases had altogether occupied some weeks. The scars which remained from the variolous eruption were quite different from those of the varioloid; those on the face still indicate their origin by their characteristic signs. The scars of the varicella, on the contrary, disappeared very soon after the disease, and those of the varioloid eruption have been lost sight of for many years, so that now no trace of either of them can be discerned.

[The author has added some rather lengthened observations; but they are not sufficiently apposite to his singular though somewhat loosely related case to render their translation necessary.]

*Medicinische Zeitung. Mai* 20, 1840.

---

### *Case of Menstruatio Recidiva.* By Dr. Petersen.

A healthy woman, aged seventy-nine, was seized on the 26th of March with uterine pains, these lasting a few days were terminated by a hemorrhagic discharge. On the 23d of April she was again affected in the same way, the discharge appearing on the 25th and continuing four days. Since that period (twelve months ago) she has regularly *menstruated.*

[There are several cases on record in which the menstrual periods have been regularly continued until seventy, eighty, and ninety years of age. It has been said (Locock) that such cases are not genuine cases of menstruation, but sanguineous discharges arising from uterine disease. But we have at this moment a lady aged sixty-two under our care. who regularly menstruates without any symptom of uterine disease. A most extraordinary case, similar to that of Dr. Petersen's, is given by Velasquez, of Tarentum, of the abbess of Monvicaro, who at the age of 100, after a severe illness, had a recurrence of the catamenia; and not only this, a new set of teeth and a fresh head of hair appeared!]

*Bibliothek for Læger. Feb.*, 1840.

---

### *Anatomico-Pathological Researches on Cirrhosis of the Liver.* By Alfred Becquerel, Paris.

The name cirrhosis was given by Laennec to a peculiar affection of the liver in which that organ appears as if filled with numerous yellow granules, which are sometimes present in such numbers that the surface of a section of the liver presents an uniform yellow colour. If this surface is carefully examined, M. Becquerel says an innumerable quantity of minute granulations may be discovered, which may be compared to the lobules of hardened and reddish-coloured fat which are commonly found in the subcutaneous cellular tissue of the thigh and leg of anasarcous subjects. These little masses are sometimes intimately united to the texture of the liver, but they are frequently separated from it by a thin layer of cellular tissue which forms a slight envelope round the globules from which they are easily detached.

The exact nature, the causes, and the symptoms of this affection are very imperfectly understood; and it is for the purpose of elucidating these points that M. Becquerel has published the present memoir. Laennec considered that in this disease a new morbid product was deposited in the liver, which he termed "cirrhose," and which he believed might be also developed in other organs of the body; appearing first in a state of crudity and afterwards softening. Andral states that the granules, in his opinion, merely arise from hypertrophy of the white or secreting part of the liver, which in the more advanced stages is accompanied with corresponding atrophy of the red or vascular tissue, giving rise to that shrivelled state of the liver generally which was described by Laennec as frequently occurring in this disease. M. Cruveilhier considers that cirrhosis consists in atrophy of a great number of the lobules of the liver, and a corresponding degree of hypertrophy of those which remain which causes the irregular granulated surface. Dr. Carswell, who gives an admirable description and delineation of this disease in the article Atrophy, in his work on Pathological Anatomy, regards it as consisting in atrophy of the lobular structure of the liver, produced by the pressure of a contractile fibrous tissue formed in the capsule of Glisson; an opinion which, as we shall find, very nearly coincides with that adopted by the author of the present paper. M. Bouillaud regards cirrhosis as a disunion of the two natural elements of the liver, the yellow granulations being nothing more than the secreting lobules undergoing disorganization in consequence of obliteration of the vascular tissue and consequent obstruction of the hepatic circulation.

M. Becquerel, previously to stating his own opinions on the nature of the morbid alteration of the liver in cirrhosis, briefly reviews the different opinions concerning the intimate anatomical structure of this organ in its natural state; and he adheres to the notion that there are two distinct substances: one yellow, which is the true secreting tissue, and the other red, which consists of the ramifications of veins and arteries. He inclines to the opinion of Cruveilhier, that each lobule possesses a spongy tissue not injectible: and thus widely differs from Mr. Kiernan, who describes the liver as being throughout essentially vascular; the lobules consisting of minute radicles of the biliary ducts surrounded by a plexus of the terminating branches of the portal veins. The latter eminent pathologist explains the differences in appearance between the red and yellow parts of the liver by the different degrees of injection of the different portions of its tissue.

Admitting, then, the presence in the liver of secreting lobules, into the interior of which no vessels enter, not even the minute ramifications of the biliary ducts, which together with the branches of the vena porta and hepatic artery form a network round the lobules without penetrating them; the lobules themselves merely consisting of a mass of albuminous corpuscules, as described by MM. Dujardin and Verger; M. Becquerel next endeavours to ascertain which of the tissues—the secreting or the vascular—is the one altered in cirrhosis, and he arrives at the conclusion that it is the latter only. He says that this tissue becomes infiltrated with a yellowish plastic or fibro-albuminous matter, which possesses the properties which characterize fibrine and albumen; for it is coagulated by heat, and by a continued application of that agent is converted into a horny substance. He considers that this effused matter is analogous in its composition to the false membranes which are developed in mucous, and more frequently serous, tissues; and he says that it undergoes the same changes.

M. Becquerel has determined that in this disease of the liver no pus globules can ever be detected in the altered structure; a very small quantity of fatty matter is likewise present, which is always abundant where pus is met with. He deduces from these facts that cirrhosis does not arise from acute inflammation: the absence of fat will also distinguish this disease from the fatty disease of the liver,* in which this organ is likewise of a yellow colour.

This fibro-albuminous matter is supposed to be deposited by interstitial infiltration in the central part of the secreting lobules, and the yellow substance of the liver becomes thus hypertrophied and causes compression and eventually atrophy of a great part of the red or inter-lobular substance. Many of the vessels and ducts are thus obliterated, and the tissue of the liver becomes difficult of injection. In the most advanced stages of this disease the effused matter loses great part of the water which entered into its composition, becomes firmer, and contracts in size. It thus undergoes the same changes as false membranes, which are at first soft and thick like white of egg, but which afterwards becomes organized and firm, and lessen in volume. From the contraction of this matter the yellow substance of the liver which was at first hypertrophied becomes atrophied, the altered lobules are diminished in volume, and many of them often coalesce, forming the patches which were described by Laennec as occurring in certain forms of this disease, and which give an irregular appearance to the surface or to a section of the liver. When this retraction has taken place, a great portion of the red or interlobular tissue of the liver no longer exists, and we only see an irregular agglomeration of yellow tubercles of different sizes, and the whole organ becomes hardened and shrivelled.

The immediate causes of this disease are completely unknown. M. Becquerel conjectures that it may arise from active and long-continued congestion of the liver, consequent upon chronic disease of the lungs or heart (with the affections of which organs cirrhosis is often complicated), or arising from other causes.

Cirrhosis never affects the liver partially, it always attacks all parts of its substance simultaneously. The gall-bladder is almost always quite natural. M. Becquerel has not made any comparative analysis of the state of the bile in this disease, but he found it vary considerably in consistence and colour.

This affection of the liver is mostly complicated with organic disease of other parts, as Bright's disease of the kidney, and various lesions of the heart and lungs; it is rarely met with by itself: M. Becquerel only found seven out of forty-two cases which he could consider as uncomplicated. He classes these complicating diseases under two heads: *First*, Chronic diseases, developed before or at the same time as the cirrhosis. *Second*, Diseases more or less acute, and probably consequences of the cirrhosis. 1. To the first class belong the following:—Lesions of the *Heart*. These are more commonly met with in connexion with cirrhosis than any other; for in forty-two subjects examined the heart was affected with chronic disease in twenty-one cases or in half; and the disease in all these instances had advanced to such a degree that it must have preceded, if it did not give rise to, the disease of the liver. The disease of the heart in these cases is often complicated with other affections, as emphysema of the lungs and chronic bronchitis, tubercular disease, or Bright's kidney.

It is exceedingly difficult to determine whether the affection of the heart gives rise to that of the liver, and if so in what manner. M. Becquerel has no doubt that the former precedes the latter in almost all cases, and supposes that it may give rise to it by keeping up a state of continued congestion of the liver. Pulmonary emphysema and chronic bronchitis, which are sometimes found accompanying cirrhosis, without disease of the heart, may be supposed to act in its production in the same manner, by obstructing the venous circulation. Cirrhosis is very rarely found in connexion with tubercular disease of the lungs. The fatty degeneration of the liver, on the contrary, is a frequent complication of consumption.

The morbid alteration of the *kidney*, known by the name of Bright's disease, frequently accompanies cirrhosis, having been met with fifteen times in forty-two cases. Some differences were observed between these cases of considerable

* See Brit. and For. Med. Rev. Vol. VIII., p. 554.

interest: thus in nine cases where cirrhosis and renal disease existed together and appeared to be of about the same standing, old organic lesions of the heart and lungs were also found, which our author considers to have been the exciting causes of the diseases of both the liver and kidneys, repeated congestion of both these organs having been occasioned by obstruction to the circulation. In another series of cases, which includes three out of fifteen, the cirrhosis seemed to be of a much older date than the affection of the kidneys, and probably gave rise to it by obstructing the circulation of the abdominal veins. In the remaining three cases the disease of the liver and kidneys seemed to be of about the same date and had been probably developed from a similar cause, the nature of which, however, was unknown.

2. In the second class may be arranged the following:—*Ascites*, which constantly accompanies cirrhosis in the more advanced stages; *Peritonitis*, producing purulent effusion and the formation of false membranes; *Pleuritis* and *hydrothorax*; *Pericarditis*—this has only been met with in a few cases; *Anasarca*—this generally occurs in complicated cases along with ascites and when the disease of the liver is in an advanced stage. Besides these, *œdema of the lungs*, *pneumonia*, and *pulmonary apoplexy* have been met with, as well as congestion of the mucous membrane of the alimentary canal; and *intestinal hemorrhage*, which must be considered as an effect of the disease of the liver, arising from the obstruction of the circulation of the blood through the vena porta.

Cirrhosis is more frequently met with in men than in women: thus of eighteen cases of the simple or uncomplicated form of this disease, twelve occurred in males and six in females. Of the whole number of forty-five cases examined by M. Becquerel, twenty-eight were found in men and seventeen in women.

With regard to the influence exerted by age, the eighteen simple cases may be thus arranged:

| | | | | | | |
|---|---|---|---|---|---|---|
| Between | 18 | and | 20 | years. | | 1 |
| ,, | 20 | ,, | 30 | ,, | | 2 |
| ,, | 30 | ,, | 40 | ,, | | 7 |
| ,, | 40 | ,, | 50 | ,, | | 3 |
| ,, | 50 | ,, | 60 | ,, | | 5 |

Thus persons between thirty and forty seem to be most liable to be attacked by cirrhosis. This disease is also met with in children; and M. Becquerel is disposed to think that it occurs more frequently in young subjects than has generally been supposed.

With regard to the symptoms produced by cirrhosis, none exist sufficiently characteristic to enable the physician to diagnosticate its presence during life. The most constant symptom of this disease in its more advanced stages is dropsy, which always commences in simple cases in the abdomen; its approach is generally unaccompanied by pain, but in a few cases the belly is the seat of pain, either with or without pressure, and the presence of peritonitis is thus indicated. Anasarca sometimes precedes the ascites, but Bright's disease of the kidney or disease of the heart are then always indicated. M. Becquerel says that the state of the urine is always peculiar in this disease; it is of a deep orange and often reddish-yellow colour, very dense, strongly acid, and deposits an unnaturally large precipitate of urate of ammonia, which is of a bright red or cinnabar colour. The causes of this state of the urine are unknown.

The course of this affection is uniform and continued, and it always proceeds more or less quickly to a fatal termination, being apparently uninfluenced by any plan of treatment.

*Archives générales de Médecine.* Avril et Mai, 1840.

---

*On the Danger of applying Leeches which have been previously used.* By M. PUCHE.

THE transmission of virus by the fangs of leeches is an important question and worthy of examination. M. Puche, physician to the hospital of Midi, has treated a patient in his wards, who affords a convincing proof of the danger of employing leeches which have been applied before. A messenger, æt. 24, was admitted into the hospital with urethritis of four months' standing, which had been recently complicated by acute inflammation of the epididymis. The epididymitis was the consequence of excessive labour, not of arrested discharge. Many applications of leeches were made to the hypogastric region for its removal. Five of the leeches applied were purchased at a low price by the nurse. Their bites inflamed, and took the aspect of Hunterian chancres. Now these syphilitic ulcers were too recent to have arisen from the same impure connexion that produced the gonorrhœa; but as it was possible that they were occasioned by the gonorrhœal matter coming into contact with the leech-bites, M. Puche, to satisfy himself, inoculated one part with the whitish discharge, and another part with the pus of the ulcers on the 28th of February, 1840. On the 4th of March the inoculation of the urethral matter had produced nothing, while that of the chancres had given an ecthymous pustule, which had the regular development of syphilitic pustules, and terminated by an indurated and coppery cicatrix. It is argued that the ulcers proceeded from the leech-bites (the leeches had certainly been employed on a syphilitic patient,) and that they had conveyed the infection from one patient to the other. The possibility of this transmission may be granted; but other experiments are necessary to prove it, and to determine whether the leeches are not destroyed by the virulent principle they imbibe; whether the poison is destroyed by them; and if so at what period do they become innocuous.

*Bulletin Général de Thérapeutique. Juillet* 30, 1840.

---

*On the Itch in Adults and in Children.* By Dr. KRAUSE, Physician to the Poor in Dantzic.

THE chief object of this paper is to prove the inseparable connexion between itch and the acari, which many have supposed to be only occasionally present in that disease. The author gives examples to prove, what we believe has not been before noticed, that the disease may exist in those who wash themselves very often or who have very tough skins without any eruption; the itching and the power of communication may be present, but no visible sign of the disorder may exist except the burrows of the insects. The following are his cases:

1. A journeyman tailor had three weeks previously slept with a comrade who had the itch. He came to me with a burrow in his left-index finger, from which I drew out an acarus, and I then examined him most carefully, but could discover no trace of any further eruption.

2. A servant-girl, the skin on whose hand and forearm was scarcely inferior to leather in colour and thickness, complained of itching in it. By the side of the finger I discovered seven burrows, out of which I extracted three acari; but there was not a pimple or a vesicle to be found on the whole body.

3. A washerwoman, the mother of a numerous family, remarked that her two youngest children were constantly scratching themselves and had an eruption on their hands. She showed me the children, and they had distinct itch, but she denied having it herself; she allowed, however, that she had sometimes felt an itching in the fingers and arms, but she had never remarked any vesicle. This statement was correct, for on the fingers, which had a kind of varnished appearance from the constant action of the hot water and the soap-ley, I could find no eruption, though there were several burrows, from which I pulled out four acari. On further questioning it appeared that the woman had long noticed these, but had thought nothing of them because there were no vesicles.

There are only two cases, the author says, in which one would not have a right to maintain that itch did not exist, when neither burrows nor acari can be found; namely, quite at the beginning of the disease, and when treatment has been employed for some days and the skin is partially scaling off, so as to destroy the burrows and the acari, but not the brood, which may soon after generate a new eruption.

If one has the luck to draw an acarus by daylight, one may easily be sure of his existence by cracking him on the thumb-nail; he makes a noise just like, only weaker than, that which his near neighbbour the fair hexaped makes in the same circumstances. But if one brings him close to the flame of a candle he bursts with a distinct snap.

*Casper's Wochenschrift. Juli* 25, 1840.

---

*On the Employment of Lactate of Iron.* By MM. GELIS and CONTE.

AFTER stating some objections to the preparations of iron in common use, the authors give their reasons for supposing that the lactate of the protoxide of iron is superior to all other ferruginous preparations. These are that lactic acid is universally distributed throughout the body; and that all authors have endeavoured to administer iron in the form most easily soluble in the gastric juice. Berzelius, Tiedemann, and Gmelin, Dumas, Leuret, and Lassaigne, have shown that the gastric juice has sufficient lactic acid to account for its dissolving property. They find that the most useful preparations of iron are those most soluble in lactic acid and the reverse, and therefore consider it probable that iron after administration is converted into lactate of iron. Thus they were led to give the lactate direct.

M. Bouillaud and other members of the academy administered this preparation in twenty-one cases of anemia and chlorosis; but though they speak favorably of it, it did not appear to them to possess any decided advantage over other soluble preparations of iron.

*Bulletin de l'Académie Royale de Médecine. Fevrier* 29, 1840.

